# Quinolone-resistant *Escherichia coli* at the interface between humans, poultry and their shared environment- a potential public health risk

**DOI:** 10.1186/s42522-023-00079-0

**Published:** 2023-02-28

**Authors:** Mabel Kamweli Aworh, Jacob K. P. Kwaga, Rene S. Hendriksen, Emmanuel C. Okolocha, Erin Harrell, Siddhartha Thakur

**Affiliations:** 1 Department of Veterinary and Pest Control Services, Federal Ministry of Agriculture and Rural Development, Abuja, Nigeria; 2 Nigeria Field Epidemiology and Laboratory Training Programme, Abuja, Nigeria; 3 Department of Veterinary Public Health and Preventive Medicine, Ahmadu Bello University, Zaria, Nigeria; 4 Department of Population Health and Pathobiology, College of Veterinary Medicine, North Carolina State University, Raleigh, NC USA; 5 Reference Laboratory for Antimicrobial Resistance, WHO, FAO, National Food Institute, Technical University of Denmark, Kgs. Lyngby, EU Denmark

**Keywords:** *Escherichia coli*, Plasmid-mediated quinolone resistance, Plasmid-mediated colistin resistance, Poultry workers, Chicken, Poultry environment, Nigeria

## Abstract

**Background:**

Commensal *Escherichia coli* residing in the guts of humans and animals are reservoirs of multidrug resistance (MDR) genes, including quinolone resistance genes, in humans and poultry. This study aimed to characterize quinolones resistance in *E. coli* recovered from poultry workers, chickens, and poultry farm/market environments in Abuja, Nigeria.

**Methods:**

This was a cross-sectional study conducted between December 2018 and April 2019 comprising poultry workers, chickens and their poultry farm/market environments. This study characterized *E. coli* isolates from stool, faecal and environmental samples using antimicrobial susceptibility testing and whole-genome sequencing methods. Core-genome multilocus sequences-based phylogeny was used to determine the relatedness between quinolone-resistant *E. coli* isolates. Data were analyzed using descriptive statistics.

**Results:**

Of 110 *E. coli* isolates, quinolone-resistant phenotypes were observed in 68.2% (*n* = 75) isolates. Whole-genome sequencing detected plasmid-mediated quinolone resistance (PMQR) genes in 63.6% (*n* = 70) isolates. The most prevalent PMQR gene detected in 56 of these 70 *E. coli* isolates was *qnrS1*, followed by *qnrB19* in 14 isolates and *aac(6’)-lb-cr* in two isolates. Fifteen ciprofloxacin and 19 nalidixic acid-resistant isolates respectively showed double mutations in the quinolone-resistance determining regions (QRDRs) of *gyrA*, with single or double mutations in *parC*, and a single mutation in *parE*. The most prevalent amino-acid substitutions observed were S83L + D87N in *gyrA* (46.5%, *n* = 20), S80I in *parC* (51.2%, *n* = 22) and S458A in *parE* (14%, *n* = 6). About 2.9% (2/70) of PMQR isolates were extended-spectrum beta-lactamase (ESBL) producers while 2.9% (2/70) had plasmid-mediated colistin resistance (PMCR) genes.

**Conclusions:**

PMQR genes were prevalent in *E. coli* isolates recovered from healthy humans, chickens and poultry farm/market environments. PMCR genes (*mcr-1.1*) occurred in PMQR-positive isolates recovered from manure and drinking water originating from poultry farm/market environments. It was found that the gene encoding ESBL coexisted with *qnrS-*positive isolates of human and avian origin. Horizontal transfer of PMQR genes among *E. coli* isolates in the human-poultry-environment interface has public health implications for the spread of antimicrobial resistance. Relevant government agencies should enforce regulations to restrict the use of critically important antimicrobials in poultry production.

## Background

Globally, commensal *E. coli* is known as an inhabitant of the intestinal microflora of warm-blooded humans and animals including poultry [1–3]. Although commensal *E. coli* is known not to be harmful to the host, studies have reported that the bacteria can acquire resistance and become a reservoir for multidrug resistance (MDR) genes including plasmid-mediated quinolone resistance (PMQR) genes [4, 5], hence serve as a useful indicator organism for measuring antimicrobial resistance (AMR) [4, 6, 7]. In most developing economies including Nigeria, the lack of enforcement on access, sale, and use of antimicrobials in humans and food-producing animals has resulted in the abuse of antimicrobials [8–10].

Fluoroquinolones are one of the available therapeutic options for *E. coli* infections in humans [11]. Quinolones and colistin have been classified as the highest-priority important antimicrobials for use in humans by the World Health Organization (WHO) because a strong correlation exists between use and an increase in drug resistance [11]. Several studies have reported resistance to ciprofloxacin and nalidixic acid in *E. coli* isolates recovered from food-producing animals particularly poultry as well as in humans and the environment [12–16]. However, the emergence of quinolone resistance has been attributed to the fact that fluoroquinolones are one of the most commonly prescribed antimicrobial classes in humans and food-producing animals in Nigeria [17–19]. Previous studies have reported that there is a paucity of information on PMQR and its importance in Africa, although resistance has been reported to evolve quickly in Nigeria [20]. *E. coli* isolates have enzymes important for bacterial replication which function as target sites for fluoroquinolones. These enzymes include DNA gyrase which acts as the primary site (*gyrA* and *gyrB*) and topoisomerase IV as the secondary site (*parC* and *parE*) [14]. Previous studies reveal that chromosomal mutations which modify the target enzymes are responsible for quinolone resistance in *E. coli* strains [21, 22]. Although quinolones and colistin have been classified as antimicrobials of last resort, co-occurrence of PMQR and plasmid-mediated colistin resistance (PMCR) have been reported in *E. coli* isolates recovered from poultry farm environments [23]. Evidence shows that PMQR genes may be found on conjugative plasmids with the ability for horizontal transfer between bacteria [24, 25]. Few studies have reported that plasmids carrying PMQR genes can harbor genes that can confer resistance to other antimicrobial agents including colistin [23, 24, 26].

Hypothesis: Poultry harboring PMQR *E. coli* isolates can become potential sources of horizontal transfer of resistance genes to poultry workers as well as to the poultry farm or market environments. We characterized quinolones resistance in commensal *E. coli* recovered from poultry workers, chickens, and selected poultry farms/market environments in Abuja, Nigeria using disk diffusion, broth microdilution, and whole-genome sequencing.

## Methods

### Research design

This cross-sectional study was conducted in Abuja, North Central Nigeria from December 2018 – April 2019. The study population comprised poultry farmers, poultry sellers, and chickens in farms/markets as well as the poultry farm/market environments. The sample size formula for cross-sectional studies was used to calculate the minimum sample size of 384 for the study with the following assumptions: expected proportion of 50%, with a precision of 5% and type 1 error of 5% [27]. A total of 429 samples were randomly selected from 52 commercial poultry farms and eight poultry markets in Abuja Municipal and Kuje Area Councils of the Federal Capital Territory, Abuja. The samples comprised 122 freshly collected human stool samples from poultry workers, 111 chicken faecal samples, and 196 environmental samples (poultry litter and drinking water), respectively. After collection, all samples were transported in ice to the National Reference Laboratory, Gaduwa, Abuja for processing within hours of collection.

### Isolation and identification of bacterial strains

A total of 110 *E. coli* strains were isolated from stool samples of apparently healthy individuals working with chickens in farms or markets, faecal samples of chickens, poultry litter, and poultry drinking water from the farm or markets as described previously [28]. Briefly, about one gram of human stool, one gram of chicken faeces and 30 g of poultry litter, respectively, were placed in 9 mL of buffered peptone water (BPW) and incubated for 24 h at 37 °C. Thereafter, a 10µL loopful of BPW was plated on MacConkey agar plates and incubated for 24 h at 37 °C. Suspected pink to red *E. coli* colonies were transferred on Eosin Methylene Blue (EMB) agar plates for 24 h at 37 °C. Subsequently, isolates with a greenish metallic sheen were plated on Tryptic Soy agar for biochemical tests (indole, methyl red, Voges-Proskauer and Citrate utilization) and confirmed using commercially available kits, Microbact GNB 24E (Oxoid, UK) following the manufacturer’s instructions. For each 100 ml of a drinking water sample, the membrane filtration technique was employed using single sterile 0.45 µm pores filter disks. Thereafter the filter membranes were placed on EMB plates and incubated for 24 h at 37 °C. All *E. coli* isolates were subsequently tested as mentioned above.

### Antimicrobial susceptibility testing

The Kirby Bauer disk diffusion method was used to determine the antimicrobial susceptibility patterns of the *E. coli* isolates against a panel of 14 antimicrobial agents namely ampicillin (10 μg), amoxicillin/clavulanic acid (20/10 μg), tetracycline (30 μg), gentamicin (10 μg), cefuroxime (30 μg), streptomycin (10 μg), chloramphenicol (30 μg), nalidixic acid (30 μg), sulfamethoxazole-trimethoprim (10 μg), nitrofurantoin (300 μg), ceftriaxone (30 μg), imipenem (10 μg), ceftazidime (30 μg) and cefotaxime (30 μg) as previously described [28]. Furthermore, the minimum inhibitory concentrations (MIC) against a panel of 16 antimicrobial agents for all *E. coli* isolates were determined by broth microdilution assay methods using the Gram-negative Sensititre™ (ESBL) plate according to the manufacturer’s instructions. These antimicrobials comprised ampicillin, cefazolin, ceftriaxone, cefepime, cefoxitin, cefotaxime, cefpodoxime, ceftazidime, cephalothin, ciprofloxacin, gentamicin, imipenem, meropenem, piperacillin/tazobactam, cefotaxime/clavulanic acid and ceftazidime/clavulanic acid. The recommendations of the Clinical and Laboratory Standards Institute (CLSI) M100 31^st^ Edition were used to interpret the results [29]. *E. coli* ATCC 25922 and *K. pneumonia*e ATCC 700603 were used for internal quality control. Multidrug resistance (MDR) was defined as resistance to three or more drug classes.

### Whole-genome sequencing (WGS) of *E. coli* isolates

The DNA of all the *E. coli* isolates was extracted using the Lucigen MasterPure™ Gram Positive DNA Purification Kit (ScienceVision, Selangor Darul Ehsan, Malaysia) following the Whole-Genome DNA Isolation for Gram-Negative Bacteria protocol according to the instructions of the manufacturer. This was followed by DNA quantification using the Qubit 4.0 Fluorometer assay kit (Thermo Fisher Scientific, Waltham, MA). Thereafter, libraries for WGS were prepared for each *E. coli* isolate using a Nextera XT DNA Library Preparation kit (Illumina Inc., San Diego, CA). For each isolate, high-quality Illumina MiSeq (Illumina Inc., San Diego, CA) deep shotgun 2 × 250 bp paired-end reads were assembled de novo into the draft genome sequence using SPAdes assembler v.3.13.1 [30].

### Characterization of antimicrobial resistance determinants of *E. coli* isolates

The in silico detection and characterization of AMR determinants was performed using the ResFinder 4.1 tool (database version 2022–04-24), which is freely available on the Center for Genomics Epidemiology (CGE) website (https://cge.food.dtu.dk/services/ResFinder/) [31]. The ResFinder tool was used to determine the acquired AMR genes and predict the chromosomal point mutations. A 90–100% identity, 60% minimum length, and 90% threshold were used to match individual genes for each isolate to an annotated resistance gene [31]. In silico analysis of the plasmid replicon types for each isolate was conducted using the PlasmidFinder 2.1 tool (2021–11-29) available on the CGE website [32].

### Determination of * E. coli* phylogroups, MLST, cgMLST and phylogeny

Multilocus sequence typing (MLST) was performed using MLST 2.0 (2022–11-14) with the *E. coli* PubMLST database (https://pubmed.ncbi.nlm.nih.gov/30345391/) [33]. The MLST analyses was based on the scheme previously described by Achtman [34] which considered allelic variations amongst seven housekeeping genes (*adk, fumC, gyrB, icd, mdh, purA,* and *recA)* to assign sequence types (STs).

We determined the phylogenetic classification of the *E. coli* genomes using ClermonTyping method as previously described [35]. The CATIBioMed (IAME UMR 1137) hosts the ClermonTyper web interface accessible at http://clermontyping.iame-research.center/. The clonal relationship between isolates was estimated by their core genome MLST (cgMLST) profile which was determined using the cgMLSTFinder 1.2 (2021–08-29) with the Enterobase scheme [36, 37].

The cgMLST-based phylogenetic tree was annotated and visualized using the FigTree version 1.4.4 tool (http://tree.bio.ed.ac.uk/software/figtree/) and interactive Tree of Life tool – iTOL version 6 (http://itol.embl.de/itol.cgi).

### Data analyses

Data were analyzed using Epi Info™ version 7.2.5.0 (https://www.cdc.gov/epiinfo/index.html). Descriptive statistics were used to summarize the data obtained. The raw reads for each *E. coli* isolate have been deposited in the National Center for Biotechnology Information (NCBI) database (Genome Trakr project) with the accession number PRJNA293225.

## Results

### Antimicrobial Susceptibility profile of quinolone-resistant *E. coli*

Overall, 110 *E. coli* isolates were recovered from 429 samples originating from 122 poultry workers, 111 chickens and 196 poultry farm/market environments. Of these, 75 (68.2%) isolates were resistant to nalidixic acid (NA) by disk diffusion method and 32 (29.1%) were resistant to ciprofloxacin (CIP) by broth microdilution method (Table 1).

**Table 1:** Prevalence of nalidixic acid or ciprofloxacin-resistant *Escherichia coli* isolates from different sources characterized by disk diffusion and broth microdilution methods

Origin	No of samples *n* = 429	No of *E. coli* isolates *n* = 110 n (%)^a^	No of NA-resistant *E. coli n* = 75 *n* (%)^b^	No of CIP- resistant *E. coli n* = 32 *n* (%)^b^
Human	122	47 (42.7)	27 (36.0)	7 (21.9)
Poultry	111	36 (32.7)	29 (38.7)	15 (46.9)
Environment	196	27 (24.5)	19 (25.3)	10 (31.3)

^a^The percentage value indicates the frequency of *E. coli* isolates from different sources. ^b^The percentage value indicates the frequency of NA (nalidixic acid)- or CIP (ciprofloxacin)-resistant isolates from the collected *E. coli* isolates using disk diffusion and broth microdilution methods. Note that all isolates that showed resistance against ciprofloxacin also showed resistance against nalidixic acid

Most of the isolates were resistant to tetracycline (98.7%), trimethoprim-sulfamethoxazole (92%), streptomycin (88%), ampicillin (82.7%) and gentamicin (68%). A majority of the isolates were however, susceptible to cefotaxime (94.6%), ceftriaxone (93.3%), cefuroxime (92%), amoxicillin/clavulanic acid (89.3%), ceftazidime (88%), and imipenem (85.3%). Moreover, 98.7% of the quinolone-resistant *E. coli* isolates recovered from the different sources were observed to be MDR i.e. resistant to three or more classes of antibiotics. The antimicrobial susceptibility profile of the 75 quinolone-resistant isolates against a panel of 14 antimicrobial agents is shown in (Table 2).

**Table 2:** Antimicrobial resistance profiles of 75 quinolone-resistant *Escherichia coli* isolates from humans, chickens, and poultry farm/market environments using the disk diffusion method

**Drug Class**	**Drug**	**Resistance breakpoint (mm)** R ≤	**Human *n* = 27 (%)**	**Chicken *n* = 29 (%)**	**Environment *n* = 19 (%)**	**Total *n* = 75 (%)**
**Tetracyclines**	Tetracycline	≤ 11	26 (96.3)	29 (100.0)	19 (100.0)	74 (98.7)
**Folate Pathway antagonists**	Sulfamethoxazole/Trimethoprim	≤ 10	25 (92.6)	26 (89.7)	18 (94.7)	69 (92.0)
**Penicillins**	Ampicillin	≤ 13	22 (81.5)	26 (89.7)	14 (73.7)	62 (82.7)
**Aminoglycosides**	Streptomycin	≤ 11	23 (85.2)	26 (89.7)	17 (89.5)	66 (88.0)
	Gentamicin	≤ 12	15 (55.6)	24 (82.8)	12 (63.2)	51 (68.0)
**Phenicols**	Chloramphenicol	≤ 12	10 (37.0)	14 (48.3)	6 (31.6)	30 (40.0)
**Nitrofurans**	Nitrofurantoin	≤ 14	5 (18.5)	11 (37.9)	6 (31.6)	22 (29.3)
**Carbapenems**	Imipenem	≤ 19	3 (11.1)	6 (20.7)	2 (10.5)	11 (14.7)
**β-lactam inhibitors**	Amoxicillin/clavulanic acid	≤ 13	2 (7.4)	5 (17.2)	1 (5.3)	8 (10.7)
**3**^**rd**^** and 4**^**th**^** Generation Cephalosporins**	Ceftazidime	≤ 17	3 (11.1)	1 (3.5)	4 (21.1)	8 (10.7)
	Cefuroxime	≤ 14	3 (11.1)	3 (10.3)	0 (0)	6 (8.0)
	Cefotaxime	≤ 22	3 (11.1)	1 (3.5)	0 (0)	4 (5.3)
	Ceftriaxone	≤ 19	3 (11.1)	1 (3.5)	1 (5.3)	5 (6.7)
**Resistance to three or more classes of antibiotics**	MDR	n/a	26 (96.3)	29 (100.0)	19 (100.0)	74 (98.7)

The resistance breakpoint for the interpretation of the disk diffusion results was obtained from the 31^st^ edition of CLSI M100 guidelines

### Characterization of PMQR *E. coli* isolates

**Fig. 1:**
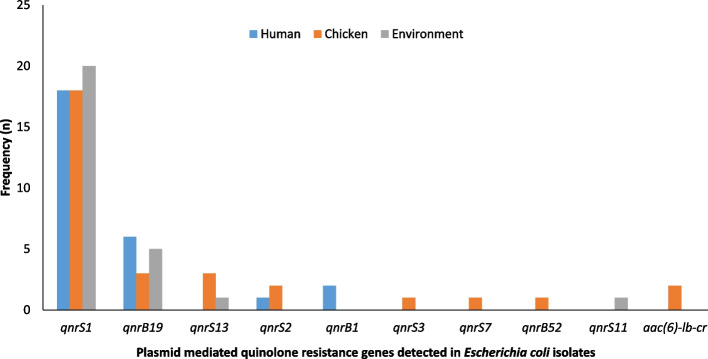
Distribution of PMQR genes in 70 *Escherichia coli* isolates originating from humans, chickens, and the poultry farm/market environment

Of the 110 *E. coli* isolates, ten distinct quinolone-resistance genes were detected in 70 (63.6%) of the isolates. Out of the 70 PMQR *E. coli* isolates, the most prevalent PMQR gene was *qnrS1*, detected in 56 (80%) isolates recovered from all sources. (Fig. 1). In addition, only 43 out of 75 phenotypically quinolone-resistant *E. coli* isolates contained PMQR genes. *E. coli* carrying PMQR genes were most often detected in chickens (34.3%) followed by humans (32.9%) and poultry farm/market environments (32.9%).

**Fig. 2:**
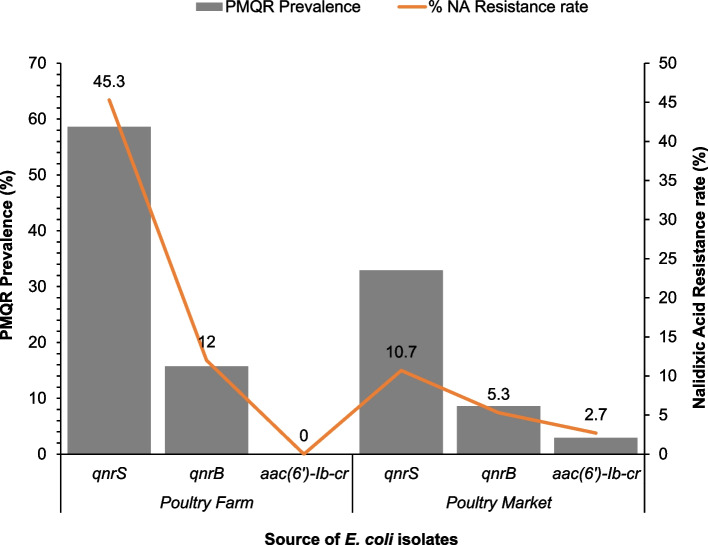
Distribution of 70 PMQR genes among 75 nalidixic acid-resistant *Escherichia coli* isolates originating from humans, chickens and poultry farms/ markets. This chart highlights the prevalence of PMQR among nalidixic acid-resistant *E. coli* isolates from all sample types originating from poultry farms/markets in Abuja, Nigeria, 2019. The prevalence of PMQR is plotted as bars on the primary axis while the nalidixic acid resistance rate in percentage is plotted as a line graph on the secondary axis. The various data points on the line graph are also displayed on the chart

Thirteen PMQR *E. coli* isolates harbored two different PMQR gene combinations namely: *qnrS1* + *qnrB19* (*n* = 9); *qnrS13* + *qnrB19* (*n* = 1); *qnrS1* + *qnrB1* (*n* = 1); *qnrS2* + *aac(6’)-Ib-cr* (*n* = 1) and *qnrB52* + *aac(6’)-Ib-cr* (*n* = 1). The nine *E. coli* isolates harboring PMQR gene combination *qnrS1* + *qnrB19* were recovered from poultry environment (*n* = 4), humans (*n* = 3), and chickens (*n* = 2). PMCR (*mcr-1.1*) gene was detected in two *E. coli* isolates originating from the poultry environment. One PMCR isolate from a water sample harbored PMQR gene combination *qnrS1* + *qnrB19* while the second isolate from a poultry litter sample harbored only the *qnrS1* gene*.* Of the 32 CIP-resistant isolates, PMQR genes were detected in 23 (71.9%) of the isolates with *qnrS*, *qnrB* and *aac(6’)-Ib-cr* genes identified in 21 (65.6%), four (12.5%) and one (3.1%) isolates, respectively. PMQR genes were detected in 48 (64%) of the 75 NA-resistant isolates with *qnrS*, *qnrB* and *aac(6’)-Ib-cr* genes identified in 42 (56%), 13 (17.3%) and two (2.7%) isolates, respectively (Fig. 2).

Two (2.9%) genes encoding extended-spectrum beta-lactamase (ESBL); *bla*_CTX-M-15_ and *bla*_CTX-M-65_ co-existed with two *qnrS*-positive isolates recovered from a single chicken originating from a poultry farm and a poultry worker at the chicken market.

### Point Mutations in the *gyrA*, *parC*, *parE,* and *pmrB* genes

Point mutations of quinolone resistance determining region (QRDR) in the DNA gyrase (*gyr*A) and DNA topoisomerase IV (*parC* and *parE*) of the *E. coli* isolates were determined by WGS. Antimicrobial susceptibility testing using the disk diffusion method showed that 75 (68.2%) isolates were resistant to NA and 43 (39.1%) of these showed point mutations. However, 55.8% (24/43) of the isolates that showed point mutations had CIP-resistant phenotypes. Overall, 43 isolates showed point mutations (Table 3), out of which 40 (93%) showed mutations in *gyrA*; 30 (69.7%) showed mutations in *parC*; and ten (23.3%) showed mutations in *parE*. One of the isolates (2.3%) showed mutations in *pmrB* which confers colistin resistance. Of the isolates that showed several point mutations, 19 (44.2%) were from chickens; 14 (32.6%) from humans, and ten (23.3%) from the poultry farm/market environments.

**Table 3:** Characterization of 43 nalidixic acid-resistant *Escherichia coli* isolates from humans, chickens, and poultry farm/market environments

Isolate number	Origin	Mutations to the QRDRs			Other mutations
		***gyrA***	***parC***	***parE***	***pmrB***
ma 001	Chicken (M)	S83L	S80I	-	-
ma 003	Environment (M)	S83L	-	-	-
ma 008	Chicken (M)	S83L	-	-	V161G
ma 010	Chicken (M)	S83L	-	-	-
ma 013	Chicken (M)	S83L + D87N	S80I	S458A	-
ma 021	Environment (M)	S83L + D87N	S80I	S458A	-
ma 024	Environment (M)	S83L	S80I	-	-
ma 038	Human (M)	S83L	-	-	-
ma 055	Chicken (M)	S83L	S80I	-	-
ma 069	Human (M)	S83A	-	-	-
ma 078	Human (M)	S83L	-	-	-
ma 105	Human (F)	S83L	-	-	-
ma 120	Environment (F)	S83L	S80I	-	-
ma 121	Chicken (F)	S83L	-	-	-
ma 126	Human (F)	S83L + D87N	S80I	L416F	-
ma 138	Human (M)	S83L + D87N	S80I	-	-
ma 140	Chicken (M)	S83L + D87N	S80I + A56T	-	-
ma 143	Human (F)	S83L + D87N	S80I	-	-
ma 146	Chicken (F)	S83L + D87N	S80I + E84G	-	-
ma 182	Environment (F)	S83L + D87N	S80I	S458A	-
ma 197	Chicken (F)	S83L + D87N	A56T + S80I + E84G	-	-
ma 218	Environment (F)	S83L + D87N	S80I	-	-
ma 233	Environment (F)	S83L	S80I	-	-
ma 234	Chicken (F)	S83L + D87N	S80I	-	-
ma 236	Human (F)	S83L + D87N	S80I	S458A	-
ma 238	Chicken (F)	S83L	S80I	-	-
ma 246	Chicken (M)	S83L + D87N	S80I	-	-
ma 249	Environment (M)	S83L	-	-	-
ma 253	Environment (F)	S83L + D87N	S80I	-	-
ma 261	Human (M)	S83L + D87N	S80I	L416F	-
ma 263	Chicken (F)	S83L + D87N	S80I	L416F	-
ma 281	Chicken (F)	S83L	-	-	-
ma 282	Environment (F)	S83L	-	-	-
ma 294	Chicken (F)	S83L	-	-	-
ma 305	Chicken (F)	S83L + D87N	S80I + A56T	-	-
ma 312	Chicken (F)	S83L + D87N	S80I + A56T	-	-
ma 330	Chicken (F)	S83L + D87N	S80I	S458A	-
ma 331	Chicken (F)	S83L + D87Y	S80I	S458A	-
ma 384	Human (F)	-	A56T	-	-
ma 390	Human (F)	-	A56T	-	-
ma 392	Chicken (F)	-	S57T	I355T	-
ma 415	Human (F)	S83L + D87N	S80I	-	-
ma 420	Human (F)	-	A56T	-	-
ma 421	Human (F)	S83L	-	-	-

^*^F – Poultry Farm; M – Poultry Market; QRDRs—quinolone-resistance determining region; Serine to Leucine at codon 83 (S83L); Aspartic acid to Asparagine at codon 87 (D87N); Serine to Alanine at codon 83 (S83A); Serine to Isoleucine at codon 80 (S80I); Alanine to Threonine at codon 56 (A56T); Serine to Threonine at codon 57 (S57T); Glutamic acid to Glycine at codon 84 (E84G); Serine to Alanine at codon 458 (S458A); Isoleucine to Threonine at codon 355 (I355T). “- “: represents the absence of mutations

There were no point mutations observed in *gyrB* as well as in the two isolates with PMCR genes. The single isolate that showed a mutation in *pmrB* (V161G) conferring colistin resistance was recovered from a chicken originating from a poultry farm.

Of 75 NA-resistant *E. coli* isolates, 43 (57.3%) showed point mutations in the topoisomerases while 48 (64%) had at least one PMQR gene. Twenty-three (30.7%) isolates showed both PMQR genes together with point mutations in the topoisomerases. Surprisingly, seven (9.3%) isolates (five from humans and two from chickens) showed neither point mutations nor PMQR genes (Table 4). The CIP MIC for these seven isolates was quite low (≤ 1).

**Table 4:** Distribution of PMQR genes and QRDR mutations among 75 NAlidixic acid resistant *Escherichia* coli isolates

	**PMQR genes**			**Mutations to the QRDRs**					
**Sample ID**	***qnrS***	***qnrB***	***aac(6')-Ib-cr***	**gyrA**	**parC**	**parE**	**pmrB**	**Location**	**Category**
ma 001				S83L	S80I			Market	Chicken
ma 003				S83L				Market	Environment
ma 008				S83L			V161G	Market	Chicken
ma 010	*qnrS*			S83L				Market	Chicken
ma 013	*qnrS*			S83L + D87N	S80I	S458A		Market	Chicken
ma 021	*qnrS*			S83L + D87N	S80I	S458A		Market	Environment
ma 024				S83L				Market	Environment
ma 038				S83L				Market	Human
ma 051	*qnrS*							Market	Environment
ma 055	*qnrS*			S83L				Market	Chicken
ma 069				S83A				Market	Human
ma 077								Market	Human
ma 078	*qnrS*			S83L				Market	Human
ma 079		*qnrB*						Market	Human
ma 088		*qnrB*						Market	Human
ma 105				S83L				Farm	Human
ma 120				S83L				Farm	Environment
ma 121								Farm	Chicken
ma 124	*qnrS*							Farm	Human
ma 125	*qnrS*	*qnrB*						Farm	Human
ma 126				S83L + D87N	S80I	L416F		Farm	Human
ma 138				S83L + D87N	S80I			Market	Human
ma 140				S83L + D87N	S80I + A56T			Market	Chicken
ma 143				S83L + D87N	S80I			Farm	Human
ma 146	*qnrS*			S83L + D87N	S80I + E84G			Farm	Chicken
ma 158	*qnrS*							Farm	Environment
ma159	*qnrS*							Farm	Human
ma162	*qnrS*							Farm	Chicken
ma 163	*qnrS*							Farm	Environment
ma 167								Farm	Chicken
ma 168	*qnrS*							Farm	Chicken
ma 172	*qnrS*							Farm	Human
ma 174	*qnrS*	*qnrB*						Farm	Chicken
ma 175		*qnrB*						Farm	Environment
ma 179	*qnrS*	*qnrB*						Farm	Chicken
ma 182				S83L + D87N	S80I	S458A		Farm	Environment
ma 183								Farm	Human
ma 197	*qnrS*			S83L + D87N	A56T + S80I + E84G			Farm	Chicken
ma 218	*qnrS*			S83L + D87N	S80I			Farm	Environment
ma 233	*qnrS*			S83L	S80I			Farm	Environment
ma 234	*qnrS*			S83L + D87N	S80I			Farm	Chicken
ma 236	*qnrS*			S83L + D87N	S80I	S458A		Farm	Human
ma 237	*qnrS*							Farm	Human
ma 238	*qnrS*			S83L	S80I			Farm	Chicken
ma 241		*qnrB*						Farm	Human
ma 244	*qnrS*							Farm	Environment
ma 245	*qnrS*		*aac(6')-Ib-cr*					Market	Chicken
ma 246		*qnrB*	*aac(6')-Ib-cr*	S83L + D87N	S80I			Market	Chicken
ma 249	*qnrS*	*qnrB*		S83L				Market	Environment
ma 252	*qnrS*	*qnrB*						Farm	Environment
ma 253	*qnrS*			S83L + D87N	S80I			Farm	Environment
ma 257								Market	Human
ma 261				S83L + D87N	S80I	L416F		Market	Human
ma 263	*qnrS*			S83L + D87N	S80I	L416F		Farm	Chicken
ma 281	*qnrS*			S83L				Farm	Chicken
ma 282	*qnrS*			S83L				Farm	Environment
ma 286								Farm	Human
ma 287	*qnrS*							Farm	Chicken
ma 289	*qnrS*							Farm	Environment
ma 293	*qnrS*							Farm	Environment
ma 294				S83L				Farm	Chicken
ma 305				S83L + D87N	S80I + A56T			Farm	Chicken
ma 312				S83L + D87N	S80I + A56T			Farm	Chicken
ma 314								Farm	Human
ma 330				S83L + D87N	S80I	S458A		Farm	Chicken
ma 331	*qnrS*			S83L + D87Y	S80I	S458A		Farm	Chicken
ma 367	*qnrS*							Farm	Environment
ma 370	*qnrS*							Farm	Chicken
ma 384	*qnrS*				A56T			Farm	Human
ma 390	*qnrS*				A56T			Farm	Human
ma 392					S57T	I355T		Farm	Chicken
ma 415		*qnrB*		S83L + D87N	S80I			Farm	Human
ma 420	*qnrS*	*qnrB*			A56T			Farm	Human
ma 421				S83L				Farm	Human
ma 422	*qnrS*							Farm	Chicken

### Substitutions in topoisomerase subunits: *gyrA, parC,* and *parE*

Of the 40 isolates showing mutations in *gyrA*, 45% (*n* = 18) presented single amino acid (AA) substitution from serine to leucine at codon 83 (S83L) while 50% (*n* = 20) possessed double substitution at S83L and aspartic acid to asparagine at codon 87 (D87N). Of the 30 isolates which showed mutations in *parC*, 73.3% (*n* = 22) presented single AA substitution of serine to isoleucine at codon 80 (S80I); 10% (*n* = 3) AA substitution alanine to threonine at codon 56 (A56T); and 3.2% (*n* = 1) AA substitution serine to threonine at codon 57 (S57T). Two (6.7%) isolates possessed double substitution at S80I and A56T; 3.2% (*n* = 1) double substitution at S80I and glutamic acid to glycine at codon 84 (E84G); and 3.2% (*n* = 1) possessed triple substitution at S80I, A56T, and E84G. Lastly, one (3.2%) isolate presented single AA substitution S57T as shown in (Table 5).

**Table 5:** Distribution of substitutions in topoisomerase subunits detected in 43 PMQR *Escherichia coli* isolates from humans, chickens and poultry farm/market environments

Target Enzymes	Mutation	Nucleotide change	Amino acid substitution	*n* = 43 (%)
DNA gyrase ***gyrA***	S83L; D87N	TCG → TTG; GAC → AAC	S → L; D → N	20 (46.5)
	S83L	TCG → TTG	S → L	18 (41.9)
	S83L; D87Y	TCG → TTG; GAC → TAC	S → L; D → Y	1 (2.3)
	S83A	TCG → GCG	S → A	1 (2.3)
Topoisomerase IV ***parC***	S80I	AGC → ATC	S → I	22 (51.2)
	S80I; A56T	AGC → ATC; GCC → ACC	S → I; A → T	2 (4.7)
	S80I; E84G	AGC → ATC; GAA → GGA	S → I; E → G	1 (2.3)
	S80I; E84G; A56T	AGC → ATC; GAA → GGA; GCC → ACC	S → I; E → G; A → T	1 (2.3)
	A56T	GCC → ACC	A → T	3 (7.0)
	S57T	AGC → ACC	S → T	1 (2.3)
***parE***	S458A	TCG → GCG	S → A	6 (14.0)
	L416F	CTT → TTT	L → F	3 (7.0)
	I355T	ATC → ACC	I → T	1 (2.3)
***pmrB***	V161G	GTG → GGG	V → G	1 (2.3)

Serine to Leucine at codon 83 (S83L); Aspartic acid to Asparagine at codon 87 (D87N); Serine to Alanine at codon 83 (S83A); Serine to Isoleucine at codon 80 (S80I); Alanine to Threonine at codon 56 (A56T); Serine to Threonine at codon 57 (S57T); Glutamic acid to Glycine at codon 84 (E84G); Serine to Alanine at codon 458 (S458A); Isoleucine to Threonine at codon 355 (I355T); and *pmrB*- confers colistin resistance

Regarding *parE*, out of ten isolates that showed mutations, six (60%) showed a single AA substitution from serine to alanine at codon 458 (S458A); three (30%) showed a single AA substitution from leucine to phenylalanine at codon 416 (L416F) while one (10%) showed a single AA substitution from isoleucine to threonine at codon 355 (I355T).

Seven (16.3%) isolates with double AA substitutions in *gyrA* had point mutations in both *parC* and *parE* while one (2.3%) isolate with single AA substitution in *gyrA* had a point mutation in the *pmrB* gene, which confers colistin resistance (Table 5). Fifteen (15/32) CIP-resistant isolates showed double mutations in the QRDRs of *gyrA,* with single, double or triple mutations in *parC* and single mutation in *parE*.

### Clonal relationship of PMQR *E. coli* isolates

**Fig. 3:**
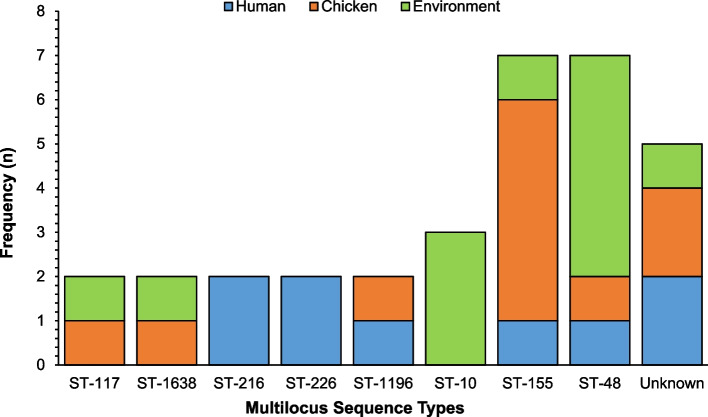
Multilocus Sequence Types for PMQR *Escherichia coli* isolates (*n* = 70) recovered from humans, chickens, and poultry environments. Each bar represents the various PMQR *E. coli* sequence types for isolates recovered from humans, chickens, and poultry farm/ market environments

The 70 PMQR *E. coli* isolates belonged to 53 different sequence types (ST), out of which 9.4% (*n* = 5) were unknown. Eight major groups were identified by in silico analysis of PMQR-isolates (Fig. 3) namely: ST-48 (13.7%; *n* = 7), ST-155 (13.7%; *n* = 7), ST-10 (5.9%; *n* = 3), ST-1638 (3.9%; *n* = 2), ST-117 (3.9%; *n* = 2), ST-216 (3.9%; *n* = 2), ST-226 (3.9%; *n* = 2), and ST-1196 (3.9%; *n* = 2).

In the ST-48 group, the most frequently represented isolates were recovered from poultry litter (5/7), followed by two isolates recovered each from a poultry farmer (1/7) and a chicken from a farm (1/7). Next was the ST-155 group from chickens at the poultry market (4/7); a chicken from a farm (1/7); a poultry farmer (1/7) and a poultry market environment (1/7). The ST-10 group carrying the *qnrS1* or *qnrS13* or the combination of *qnrS1* + *qnrB19* genes was from the poultry environment (3/3). The clustering of isolates belonging to the same phylogroup and sequence type was consistent.

The most common STs recovered from the poultry environment were ST-48 (5/7) and ST-155 (3/3). In the ST-48 group, 57.1% (*n* = 4) of the *E. coli* isolates harbored two different PMQR gene combinations namely: *qnrS1* + *qnrB19* (*n* = 3) and *qnrS13* + *qnrB19* (*n* = 1) while in ST-155 group, one isolate harbored PMQR gene combination: *qnrS1* + *qnrB19*.

Two (28.6%) PMQR isolates in the ST-155 group showed point mutations in topoisomerase genes. One isolate from a chicken at the poultry market with single AA substitutions in *gyrA* (S83L) had a point mutation in *parC* (S80I) while the remaining isolate recovered from a poultry farmer had a single AA substitution in *parC* (A56T). One (14.3%) PMQR isolate in the ST-10 group from the poultry market environment harbored a PMCR gene (*mcr-1.1*).

**Fig. 4:**
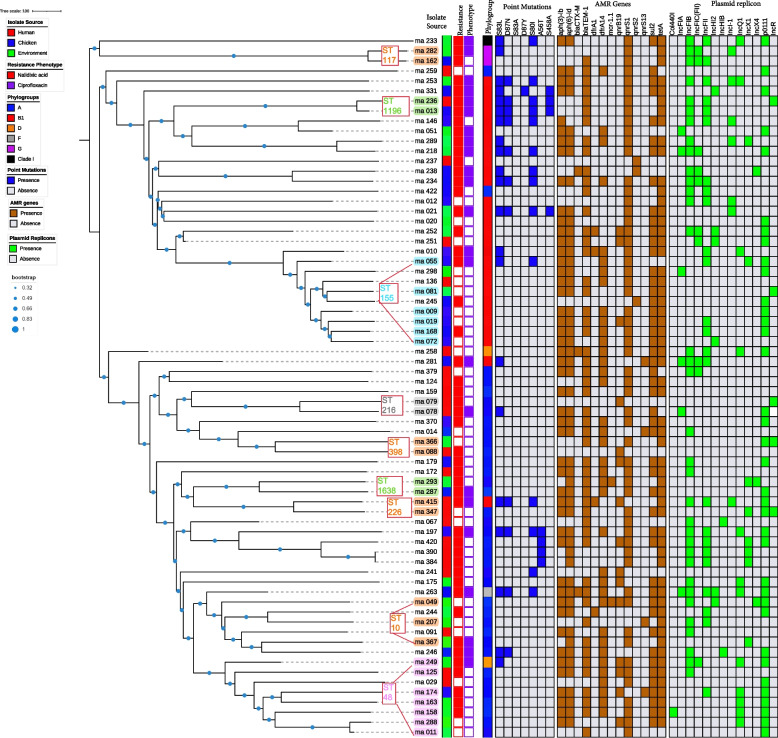
Core genome MLST-based phylogeny of PMQR *Escherichia coli* isolates (*n* = 70) from humans, chickens, and poultry environments. cgMLST-based phylogenetic tree of PMQR *E. coli* isolates visualized in Interactive Tree Of Life tool (iTOL). The clustering of isolates was found to be following the core genome. The clustering of isolates belonging to the same phylogroup and sequence type was consistent. Shown for each isolate from left to right are the source, resistance phenotype, phylogroup, point mutations, AMR genes, and plasmid replicons

The plasmid replicon profiles of the PMQR *E. coli* isolates showed that the *qnr* and *aac(6’)-Ib-cr* genes were located on different plasmids with IncFIB, p0111, IncFII, IncQ1, IncHI2, IncI1-I, IncX1, and IncX4 being the most commonly shared replicons among humans, chickens and the poultry environment (Fig. 4).

### Determination of Phylogroups

Phylogenetic classification of the PMQR isolates from humans, chickens and the poultry environment showed that 36 (51.4%) of the isolates belonged to phylogroup A, 28 (40%) to phylogroup B1, two (2.9%) to phylogroup D, two (2.9%) to phylogroup G, and one each (1.4%) to phylogroups F and clade I, respectively (Fig. 4). Of the 36 PMQR *E. coli* isolates belonging to phylogroup A, 31 (86.1%) had the *qnrS* gene; 11 (30.6%) had the *qnrB* gene while one (2.8%) had the *aac(6’)-Ib-cr* gene. Of the 28 PMQR-positive isolates belonging to phylogroup B1, 27 (96.4%) had the *qnrS* gene; five (17.9%) had the *qnrB* gene while one (3.6%) had the *aac(6’)-Ib-cr* gene.

### Antimicrobial resistance genes carried on plasmid replicon

**Fig. 5:**
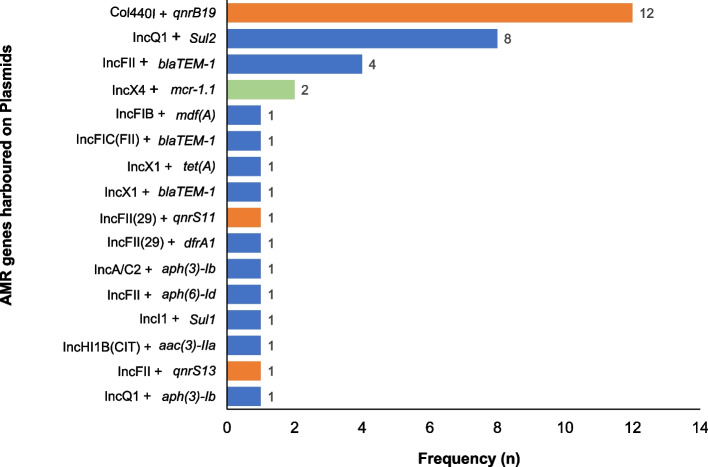
Antimicrobial resistance genes carried on plasmid replicon detected in 70 PMQR *Escherichia coli* isolates from humans, chickens, and poultry farm/market environments. Each bar represents the resistance genes carried on the plasmid replicon. The orange bars represent PMQR genes (*qnrB or qnrS*) carried on specific plasmids. The green bar represents the PMCR gene (*mcr-1.1*) carried on specific plasmids. The blue bars represent other AMR genes carried on plasmids

Thirty isolates harbored AMR genes on the plasmid replicon identified on the same assembly scaffold out of which 13 (43.3%) were recovered from the poultry farm/market environments, nine (30%) from chickens, and eight (26.7%) from humans. Five isolates harboring *qnr* genes also carried other AMR genes including the *mcr-1.1* gene on plasmid replicon (Fig. 5). These include two isolates from poultry farm/market environment: ma 049 (IncX4 + *mcr* 1.1 and Col440l + *qnrB19*) and ma 288 (IncQ + *sul2* and Col440l + *qnrB19*); two isolates from chickens: ma 174 (IncQ1 + *sul2* and Col440l + *qnrB19*) and ma 179 (IncA/C2 + *aph(3″)-lb* and Col440l + *qnrB19*) and one human isolate: ma 415 (IncFII + aph(6)-ld and Col440l + *qnrB19*). We detected IncFIB (pLF82), a phage plasmid in one PMQR isolate originating from poultry market environment harbouring *qnrS1* + *qnrB19* gene combination as well as the *mcr 1.1* gene.

## Discussion

To the best of our knowledge, this study is the first in Nigeria to report the co-occurrence of PMQR genes with PMCR genes (*mcr-1.1*) carried on the backbones of Col440I and IncX4 plasmids in PMQR *E. coli* isolates from poultry market and farm environments. This finding may not be surprising as quinolones and colistin, which are considered critically important antimicrobial agents of last resort [11], are often used in poultry production in Nigeria [38]. Over-dependence on quinolones for therapeutic purposes in human and animal populations in Nigeria may be responsible for the selection pressure favoring the development of quinolone resistance in *E. coli* isolates recovered from different sources [18, 39].

It is also interesting to note that two genes encoding ESBL; *bla*_CTX-M-15_ and *bla*_CTX-M-65_ were found to co-exist with two *qnrS*-positive isolates from apparently healthy poultry workers and chickens respectively. This is consistent with findings of other studies reporting that beta-lactamase genes are usually detected along with *qnr*-positive isolates [14, 40, 41]. A possible explanation for the very few ESBL genes detected in *qnr*-positive isolates in the present study could be because third- and fourth-generation cephalosporins are rarely used in poultry production in Nigeria.

Previous studies have reported the horizontal spread of AMR through plasmid-mediated *qnr* genes [22, 25, 42]. The most prevalent PMQR gene from all sources in this study was *qnrS1* which was found mainly in the ST-155 group while a combination of *qnrB19* and *qnrS1* was detected mainly in the ST-48 group. This is consistent with findings from similar studies conducted in Nigeria and other locations where the *qnrS1* gene was reported to be the most prevalent [14, 17, 39, 43]. The variability of resistance genes detected in the isolates in the present study is an indication of the faecal carriage of resistance determinants in apparently healthy people, chickens, and the environment.

Fluoroquinolones, which are considered the highest priority important antimicrobials based on WHO classification are indicated for use in human medicine for infections that do not respond to other antimicrobials [11]. Our study shows that a majority of the isolates harboring PMQR genes had QRDR mutations and this is in agreement with reports of other studies [42, 44]. Our data points to the fact that PMQR genes in the human-poultry-environment interface may constitute a better means of assessing quinolone exposure as opposed to chromosomal mutations, an assumption that is consistent with the literature [45].

Quinolones are reported to inhibit cell replication, transcription, and DNA repair in bacteria by disabling essential enzymes DNA gyrase and topoisomerase IV, hence mutations in these enzymes result in quinolone-resistant strains being the main mechanism of quinolone resistance in bacteria [21, 41]. Studies have shown that the *gyrA* gene mutants carrying the S83L mutation play a major role in quinolone resistance observed in *E. coli* and are significantly higher than other quinolone resistance mechanisms [15, 42, 46]. It should be noted that the results of the present study showed that 90.7% of quinolone-resistant *E. coli* strains with the *gyrA* gene showed single or double mutations at codon 83 resulting in serine to leucine substitution and/or at codon 87 resulting in aspartic acid to asparagine substitution. Previous studies also reported that double mutations in *gyrA* at codons 83 and 87 have increased [19, 45]. The results presented show that *parC* mutations encoding the amino acid substitutions S80I and E84G were detected in some PMQR *E. coli* isolates. This is consistent with the findings of other studies [41, 45, 46]. Mutations in *gyrA* and *parC* genes are the main mechanisms of quinolone-resistant *E. coli* detected from poultry workers, chickens, and poultry farm/market environments in Nigeria in the present study. Our study, however, did not observe any mutations in *gyrB*. This is consistent with the report of other studies as mutations in *gyrB* less frequently occur in *E. coli* strains probably because the phenotypic expression of *gyrB* mutation is restricted to a narrow panel of bacteria strains [47, 48].

A similar study done in Korea reported that PMQR genes were detected in CIP-resistant *E. coli* isolates and all the CIP-resistant isolates were MDR [49]. This supports our study results revealing PMQR genes in 77.3% of the CIP-resistant isolates which were also 100% MDR. Our data revealed that the majority of CIP-resistant isolates from humans, chickens, and the poultry environment harbored point mutations in either the *gyrA* or *parC* or *parE* genes. This is consistent with the reports of other studies [41, 49]. The high level of multidrug resistance observed among CIP-resistant isolates in our study is worrisome and reflects the level of antimicrobial use in poultry, hence the need for close monitoring of antimicrobial usage in production.

In the present study, the *aac(6’)-Ib-cr* gene was very rare and detected in only two isolates recovered from chickens at the poultry market. The prevalence of this gene in NA-resistant *E. coli* isolates (2.7%) was much lower than observed in the CIP-resistant *E. coli* isolates (4.5%) and in agreement with a previous study which showed that the *aac(6’)-Ib-cr* determinant could act additively in generating CIP-resistance [50]. This observation suggests that the CIP-resistant isolates are able to withstand antimicrobial pressure for longer periods allowing for point mutations to occur.

Evidence shows that most quinolone resistant *E. coli* isolates belonged to phylogroup A and B1 [41] and this supports findings of the present study which observed that majority of the fluoroquinolone resistant *E. coli* isolates belonged to phylogroup A and B1. This observation suggests that apparently healthy people and chickens may be acting as reservoir of quinolone-resistant strains and this could have occurred as a result of inappropriate use of antimicrobials in humans as well as in poultry production.

The literature has shown that horizontal transfer of PMQR genes is usually accompanied by the presence of other AMR genes [44]. The current study demonstrates the co-occurrence of PMQR genes and other AMR genes most importantly the PMCR gene (*mcr-1.1*) which were detected in two isolates. However, only one isolate showed chromosomal mutations in the *pmrB* gene, which confers colistin resistance. This is in agreement with the findings of a similar study carried out in China where both the *mcr-1* gene as well as mutations in the *pmrB* gene were detected in *E. coli* isolates recovered from food animals [51]. In a single isolate recovered from the poultry market environment, the *qnrB19*-carrying plasmid also co-harbored the *mcr-1.1* gene which was identified on an IncX4 backbone. The *qnrS1-*positive isolate recovered from poultry manure originating from the farm environment, had two plasmids; IncX4 carrying the *mcr-1.1* gene and IncX1 carrying the *tet(A)* gene. Previous studies [52–54] have identified *mcr-1* gene on the backbone of IncX4 plasmids and this is in agreement with our study results. The reason is that the IncX4 plasmids are more prevalent carriers of the *mcr-1* gene facilitating the spread of AMR genes in the poultry environments. Seven PMQR *E. coli* isolates carried more than one plasmid harboring AMR genes in the present study and this is in agreement with another study in Nigeria on quinolone-resistant isolates bearing multiple plasmids [39]. Our findings also showed that one PMQR isolate carried a phage plasmid with three resistant gene types. This is rather not surprising as AMR genes are often carried by phage-plasmids as reported by a recent study [55].

Studies have reported a clonal relationship between CIP-resistant *E. coli* isolates recovered from humans and chickens [56, 57]. However, the present study did not detect any clonal relationship between PMQR *E. coli* isolates from humans, chickens, and poultry environments. This is consistent with the findings of a similar study in the Czech Republic which reported that strains from different sources were not related [14]. The results of the present study showed that the *qnrB19* genes were located on Col440I plasmids, while *qnrS11* and *qnrS13* were located on IncFII plasmids. This suggests that although the PMQR *E. coli* isolates were not clonally related, the high rate of plasmid carriage among the isolates may have been responsible for the emergence of fluoroquinolone resistance observed at the human-animal-environment interface [39, 58]. Previous studies in Nigeria and other locations have also reported that resistant bacteria strains often carry multiple plasmids similar to the findings in the present study [39, 58].

Our study have highlighted the potential for the plasmids to facilitate the horizontal spread of *qnrS, qnrB,* and *mcr-1* genes at the human-animal-environment interface, hence should be considered a potential public health risk. The present study shows that higher levels of fluoroquinolone resistance were detected in *E. coli* isolates from chickens when compared to isolates from humans and the poultry environment and this is consistent with the literature [25]. This calls for close surveillance and monitoring of the use of fluoroquinolones as well as other critically important antimicrobials in poultry production.

## Conclusions

PMQR *E. coli* isolates were prevalent amongst apparently healthy individuals, chickens, and the poultry farm/market environments in Abuja. ST-155 and ST-48 were the most prevalent STs detected in humans, chickens, and the poultry farm or market environments in this study. PMCR genes carried on IncX4 plasmids and genes encoding for ESBLs were detected in PMQR isolates. Horizontal transfer of PMQR genes among *E. coli* isolates at the human-poultry-environment interface has public health implications for the spread of AMR. The relevant government ministries and agencies should therefore enforce regulations necessary to restrict the use of critically important antimicrobials in poultry production in Nigeria.

## Data Availability

The datasets used and analyzed during the current study are available from the corresponding author upon reasonable request. All data generated or analyzed during this study are also included in this published article [and its supplementary information files]. The datasets generated and/or analysed during the current study are also available in the National Center for Biotechnology Information (NCBI) repository (Genome Trakr project) with the accession number to the dataset PRJNA293225.

## Abbreviations

AMR: Antimicrobial resistance
CGE: Center for Genomics Epidemiology
CIP: Ciprofloxacin
CLSI: Clinical and Laboratory Standards Institute
DNA: Deoxyribonucleic acid
ESBL: Extended-Spectrum Beta-Lactamase
FCT: Federal Capital Territory
FDA: United States Food and Drug Administration
MDR: Multi-drug Resistance
MIC: Minimum Inhibitory Concentration
MLST: Multi-locus Sequence Typing
NCBI: National Center for Biotechnology Information
NA: Nalidixic Acid
PMCR: Plasmid-mediated colistin resistance
PMQR: Plasmid-mediated quinolone resistance
QRDRs: Quinolone-Resistance Determining Regions
ST: Sequence Type
WGS: Whole-genome Sequencing
WHO: World Health Organization
